# Bill Redness Is Positively Associated with Reproduction and Survival in Male and Female Zebra Finches

**DOI:** 10.1371/journal.pone.0040721

**Published:** 2012-07-12

**Authors:** Mirre J. P. Simons, Michael Briga, Egbert Koetsier, Remco Folkertsma, Matthias D. Wubs, Cor Dijkstra, Simon Verhulst

**Affiliations:** Behavioural Biology, Centre for Life Sciences, University of Groningen, Groningen, The Netherlands; Utrecht University, The Netherlands

## Abstract

Sexual traits can serve as honest indicators of phenotypic quality when they are costly. Brightly coloured yellow to red traits, which are pigmented by carotenoids, are relatively common in birds, and feature in sexual selection. Carotenoids have been linked to immune and antioxidant function, and the trade-off between ornamentation and these physiological functions provides a potential mechanism rendering carotenoid based signals costly. Mutual ornamentation is also common in birds and can be maintained by mutual mate choice for this ornament or by a correlated response in one sex to selection on the other sex. When selection pressures differ between the sexes this can cause intralocus sexual conflict. Sexually antagonistic selection pressures have been demonstrated for few sexual traits, and for carotenoid-dependent traits there is a single example: bill redness was found to be positively associated with survival and reproductive output in male zebra finches, but negatively so in females. We retested these associations in our captive zebra finch population without two possible limitations of this earlier study. Contrary to the earlier findings, we found no evidence for sexually antagonistic selection. In both sexes, individuals with redder bills showed higher survival. This association disappeared among the females with the reddest bills. Furthermore, females with redder bills achieved higher reproductive output. We conclude that bill redness of male and female zebra finches honestly signals phenotypic quality, and discuss the possible causes of the differences between our results and earlier findings.

## Introduction

Sexual traits can serve as indicators of quality and require costs to facilitate honest signalling [1], [2]. Red and yellow secondary sexual traits are found throughout vertebrates and are relatively common, especially in birds [3]. These traits have in some species been shown to feature in sexual selection (in birds e.g. [4]–[7]) and are of specific interest because in most birds they are pigmented by carotenoids [8]. In search of the costs maintaining honest advertisement of quality via yellow and red traits, carotenoids have been linked to antioxidant and immune status signalling [9]–[11]. Carotenoid-dependent traits may therefore signal phenotypic quality by advertising the ability to allocate carotenoids away from physiological functions towards sexual colouration.

Ornamentation of both sexes is also relatively common in birds. Mutual mate choice can maintain the ornamentation of both sexes [12]. Or it can be maintained via a correlated response to selection on the other sex [12], which can cause intralocus sexual conflict [13]–[15]. Most genes are carried across generations in both males and females, but the selection pressures acting on these genes can differ in strength and even in sign between the sexes, i.e. sexually antagonistic selection. For sexual traits there are few examples of sexually antagonistic selection [16]–[18] and to our best knowledge there is only one example for carotenoid dependent ornaments: bill redness of the zebra finch (*Taeniopygia guttata*) was positively related to survival and reproductive success in males, but negatively so in females [18] (but note that the survival relationship was non-significant in males). Given that the genetic correlation of bill redness is high (*r* = 0.93; [19]), intralocus sexual conflict is plausible.

Zebra finch bills derive their red colour from carotenoids and males have redder bills than females [20], [21]. Within males, bill redness reflects recent environmental [22] and immunological challenges [23]–[25], and correlates positively with immune functioning [26], [27]. These signalling attributes of bill redness may be why there is female preference for this trait [4]. In contrast, male mate choice in relation to female bill colouration has been little studied [4] and relatively little is known about the possible signalling value of female bill colouration. Two studies reported females with redder bills to deposit more carotenoids in their eggs [28], [29], which is associated with increased hatching success [29]. This suggests that also in females redder bills may be associated with higher phenotypic quality.

We tested the associations of female and male bill colouration with reproduction and survival, as did Price & Burley [18], but our study differs from theirs in two main aspects. Firstly, in the study of Price & Burley the birds were reproducing, which may have confounded the estimated association of bill colour with survival when bill colour affects reproduction and reproduction in turn affects survival. We therefore examined the association between bill colour and survival in single sex aviaries, in which birds could not reproduce. In mixed sex aviaries we examined the relationship between bill colour and reproductive success. Secondly, Price & Burley selected birds with extreme bill colours for their study, which can lead to erroneous conclusions when the associations of bill colour with survival and reproduction are not linear. We therefore did not select particular phenotypes for our study, and thus also included the intermediate phenotypes. Contrary to the results of Price & Burley we found no evidence for sexually antagonistic selection: individuals with redder bills of both sexes showed higher survival, and females with redder bills achieved higher fledgling production. Our findings thus substantiate signalling of physiological state by male zebra finch bill colouration and we show that it does so similarly in females.

## Methods

### Bill Colour Measurement

Bill colour measurements were performed using digital photography (Sony DSC-F707). Pictures were taken of the top of the bill in controlled light conditions, on a Kaiser photography table equipped with four Philips Photocrescenta 150 watt light bulbs, with manually fixed camera settings. Digital cameras often do not respond linearly to the amount and spectral properties of light [30], [31]. We corrected for this using a calibration set of colour patches (Munsell glossy finish collection, with published spectra from the Joensuu Spectral Database, http://cs.joensuu.fi/~spectral/databases/. Accessed 2012 June 17) to obtain a simulated reflectance spectrum from the digital images using Wiener estimation [32]. This methodology uses *a priori* information on the spectral reflectance of training objects (e.g. Munsell patches) captured by the digital camera RGB response (i.e. the sensors in the digital camera with spectral sensitivity to “red”, “green” and “blue”) to create an estimation matrix using Wiener estimation [32], via cross-correlation between the obtained RGB values of each patch and the known corresponding spectral reflectance of the training objects. By using not only the single RGB values, but also their polynomials an improved fit to the original spectra can be obtained [32]. We used 3^rd^-order polynomials of the obtained RGB values as input. The estimation matrix can then be used when capturing other objects than the training set to obtain simulated spectra. We did this per pixel of the bill and averaged these simulated spectra to obtain the simulated spectrum across the bill. These spectra are thus corrected for non-linearity in the response of the digital camera to light given that the estimation matrix is derived from known spectra of training objects.

**Figure 1:**
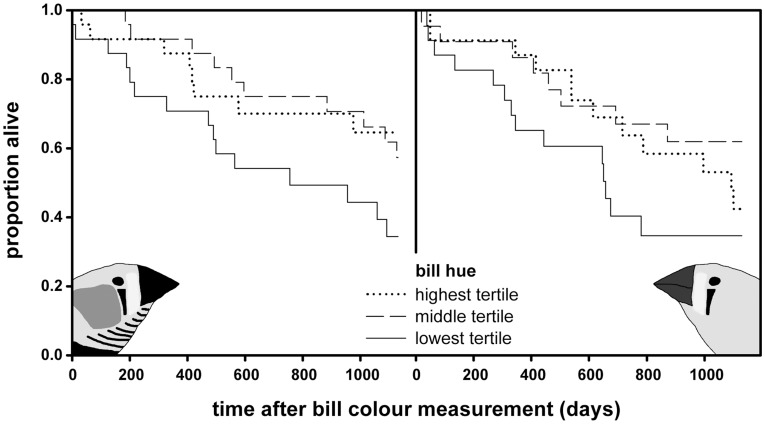
Bill hue and survival. Survival of males (left panel) and females (right panel) in relation to bill hue categories (tertiles). Note that data are shown for bill hue tertiles but bill hue was entered as continuous variable in the analyses. In both sexes individuals with low redness survive worst. In females a quadratic relationship of survival with bill hue was detected (see main text).

The spectra we obtained showed a characteristic profile for red traits: little reflection from blue toward green, increasing reflection and levelling off in the red part of this spectrum (i.e. a sigmoid shape). From this spectrum we calculated the inflection point, as a measure of hue, using non-linear fitting of a 4-parameter sigmoid curve. Chroma of the bill was calculated as the summed reflectance between 600–700 nm divided by the summed reflectance of 380–700 nm. The bill was selected automatically from each picture using cluster analysis, which was manually checked and corrected for any inaccurate selections (which occurred in <1% of the pictures). All these procedures were implemented in Matlab software (code available upon request).

**Table 1:** Proportional hazard models including both sexes.

model	parameter	estimate	s.e.	p value
without quadraticterm	bill hue	−0.090	0.026	0.00029
	sex	−0.21	0.25	0.39
	age at measurement	0.00097	0.00041	0.015
	sex X bill hue(omitted)	−0.0059	0.051	0.91
with quadratic term	bill hue	−0.037	0.037	0.32
	bill hue^2^	0.024	0.0066	0.00027
	sex	0.45	0.39	0.25
	age at measurement	0.0010	0.00042	0.013
	sex X bill hue	−0.10	0.071	0.14
	sex X bill hue^2^	−0.031	0.010	0.0023

Both chroma and hue measures were highly repeatable as estimated in a separate set of male and female birds of which we took two pictures a minute apart (hue: *r* = 0.997; chroma: *r* = 0.990; N = 30). Additionally we validated our method in this set of birds from which we obtained simulated reflectance spectra from photographs and reflectance spectra assessed with a spectrophotometer (BLK-C-100 spectrophotometer, SL4-DT (Deuterium/Tungsten) light source, R600-8-UV-VIS reflectance probe, StellarNet, FL). Estimates of both hue and chroma correlated strongly between both methods (hue: *r* = 0.92, N = 31; chroma: *r* = 0.77, N = 31). Chroma and hue covaried strongly in both directly measured (*r* = 0.88, N = 31) and simulated spectra (*r* = 0.96, N = 31). In the following we will present the results based on the measure of hue only. Analyses with chroma as dependent gave qualitatively the same results. Moreover, the majority of previous studies on zebra finch bill colouration used a Munsell colour chip system which is primarily based on hue [26], [33]. As a control for ambient and technical conditions in which the photographs were taken we included the yellow patch of a Kodak colour chart in each picture and extracted hue from this patch in the same way as for the bills. When light conditions or camera sensitivity would change, due to a factor we could not control, this will affect both the colour of the bill and the patch in the same picture. In none of the analyses was the hue measured from the Kodak chart correlated with the hue of the bill in the same picture (p>0.36).

**Table 2:** Proportional hazard model within males.

parameter	estimate	s.e.	p value
bill hue	−0.1	0.034	0.0056
bill hue^2^ (omitted)	−0.0067	0.0076	0.38
age at measurement	0.00066	0.00061	0.28

### Survival

Birds were housed in four outside aviaries (L * W * H: 320 * 150 * 225 cm), two with males (N = 72, 36 per aviary) and two with females (N = 68, 32 and 36 per aviary). Before the experiment started individuals were kept in unisexual groups of similar density as in the experimental setting and the birds had no breeding experience. Food (tropical seed mixture), water, grit and cuttlebone were provided *ad libitum*. In addition the birds received fortified canary food (“eggfood”, by Bogena, Hedel, the Netherlands) in weighed portions (0.42 gram/bird, 3 times a week; control treatment as described in [34]). All bill colouration measures were taken in November 2008, after which survival was monitored till December 2011. During this period new birds were introduced into the aviaries replacing individuals that had died, to maintain a relatively constant density throughout the experiment. This experiment started in December 2007, but due to low mortality in the first year and addition of birds in 2008, our sample size to assess correlates with survival was largest in 2008. Mortality (82 cases) was recorded daily and was analysed using proportional hazards models (using the Survival package in R, function ‘coxph’ [35]). We tested for violations of the proportional hazards assumption using the function ‘cox.zph’ and by scaled Schoenfeld residual plots. We detected no violations of this assumption. Deaths, which occurred within 48 hours after handling for experimentation (N = 9), or birds that were terminated for various welfare reasons (N = 6) and birds still alive were censored. Note, when both these categories of deaths were treated as natural deaths, this did not qualitatively change the results. Parameters included in the model were: aviary (as random term, using the function ‘frailty’), age at the time of bill measurements (mean age = 659± S.D. 329 days, range = 151–1028 days), sex, bill hue (mean centered per sex), bill hue squared and bill hue interactions with sex. In this study the rearing brood sizes of the birds were either standardised to 2 or 6 [36]. Although this did not affect either survival (when included as factor in the full model, p = 0.55) or bill colour (p = 0.97), brood size was retained in the proportional hazards models as strata. Age at the time of bill measurement was also not related to bill colouration (p = 0.66). These birds are the control treatment of a larger experiment [34], [36], in which context the birds were blood sampled for 2–3 times per year and respirometry measurements were taken 1–2 times a year, but otherwise these birds were left undisturbed.

**Table 3:** Proportional hazard models within females.

model	parameter	estimate	s.e.	p value
all females	bill hue	−0.04	0.038	0.32
	bill hue^2^	0.026	0.0076	0.00081
	age at measurement	0.0013	0.00058	0.028
females with hue < optimum	bill hue	−0.26	0.093	0.0051
	age at measurement	0.0028	0.001	0.0043
females with hue > optimum	bill hue	0.22	0.15	0.13
	age at measurement	0.00015	0.00079	0.85

**Figure 2:**
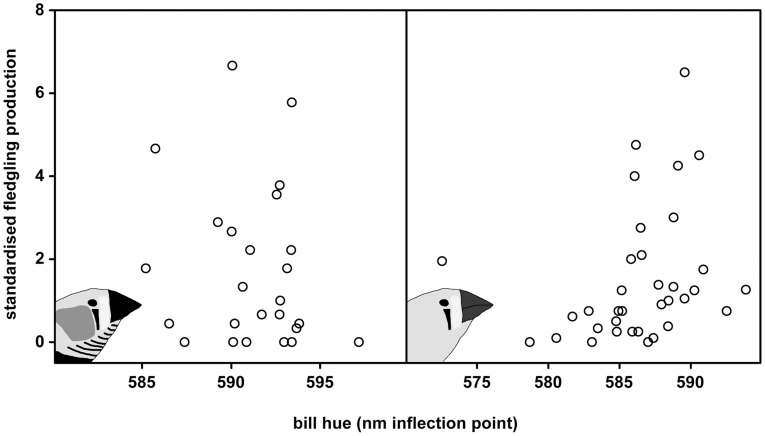
Bill hue and fledgling production. Fledgling production (standardised by dividing it by the median fledgling production per batch, thus not corrected for differences in longevity; see main text) of males (left panel) and females (right panel) in relation to bill hue. Only in females was redder bill hue significantly associated with reproductive success (see main text).

### Reproduction

This experiment was initiated in April 2009, when a mixed-sex group of adult zebra finches, previously housed in unisexual groups of similar density as in the experiment and thus without previous breeding experience, was housed in two outdoor aviaries (dimensions as in the survival measurements). Reproduction was facilitated by providing a surplus of nest boxes (20) per aviary, and nest material (hay). Offspring were removed from the aviaries at around 35 days of age, when they are usually nutritionally independent. The food regime was essentially the same as in the survival measurements, except that here egg food was provided *ad libitum*. Also in this set the birds were blood sampled for 2–3 times per year and respirometry measurements were taken 1–2 times a year in the context of other experiments, but otherwise left undisturbed. We investigated the relationship between initial bill colour and subsequent fledgling production. Bill colouration was measured of two batches of females (mean age = 415± S.D. 110 days, range = 182–737 days), before they were introduced to the aviaries in spring about one year apart (April 2009, N = 22; June 2010, N = 13). Follow up consisted of two subsequent summers for each batch (2009–2010 and 2010–2011) in which parentage was assessed by observations of chick feeding through one-way mirrors. Parentage of clutches that did not hatch was thus not assessed. Individual birds were identified by the use of colour bands (colours used: black, cyan, green, white, yellow; band colour was not associated with either reproduction (Χ^2^(4)<8.25, p>0.08) or bill hue (Χ^2^(4)<5.87, p>0.21) as tested within both sexes). Bill colour did not differ between batches (t = 0.47, df = 17, p = 0.68), but total fledgling production, broods produced and fledging per brood within the two breeding seasons of follow up differed between batches (all were higher in batch 2, p<0.05, table S1) and were left-skewed (but not Poisson distributed). Therefore we standardised these measures by dividing them by their median per batch. Mortality occurred and therefore longer-lived females had a wider window of opportunity to reproduce. To correct for this we divided fledgling production by the number of days available for breeding and further standardised this by dividing it by its median per batch. Days available for breeding was defined as the part of the year at which other birds had nestlings and when the focal bird was alive. These two relationships were assessed for significance using rank correlations. In a similar fashion we analysed correlates of male (N = 25, mean age = 674± S.D. 334 days, range = 370–1384 days) bill colour for which we only had measurements of the first batch. Pair formation was investigated in the first batch, because in this group information on bill colouration was available for both sexes. Only the first pair-bond that resulted in hatchling production in the first breeding season was considered, to avoid complication of re-pairing after deaths of partners and unknown bill coloration of males introduced in the second breeding season, and we examined whether bill colouration influenced the likelihood of pair formation. During their rearing all the birds entered in the aviaries were allowed imprinting opportunity on adults of both sexes for at least 100 days after birth, which may be important in shaping to what extent zebra finches use bill colouration in mate choice [4]. Because we did not assess extra-pair paternity, which can be as high as 29% in this species in aviary contexts [37], [38], the results on male reproduction are considerably less reliable than those on females.

### Ethics Statement

The research presented here has been approved by the animal welfare ethics committee of the University of Groningen (according to Dutch law), under license number 5150.

## Results

### Survival

Survival of individuals with redder bills was higher (figure 1, table 1; negative estimates indicate lower risk of death), and equally so in both sexes as indicated by the non-significance of the interaction between sex and bill hue (table 1). To investigate whether the observed relationship was linear we additionally tested for quadratic associations of bill hue with survival. The interaction of this quadratic term with sex was significant (table 1). Within males only the linear term was significant (table 2), whereas within females we detected a significant quadratic term (table 3, figure S1). The optimum of this quadratic relationship is 0.74 nm above the female average (mean = 583.3± S.D. 4.8) of bill hue. To test for negative survival selection we split the dataset into bill hue below and above this estimated optimum. In females showing redder bills than the optimum we did not detect significant negative survival selection with respect to bill hue (table 3). However in females with bill hue less red than the estimated optimum we found higher survival with increasing bill hue (table 3). As expected, higher age at measurement was associated with increased risk of death (tables 1, 2, 3).

### Reproduction

Fledgling production increased with bill redness in females (figure 2 right panel, *r*_s_ = 0.46, p = 0.005). This effect was not solely due to a higher survival rate of redder females, because it remained significant when fledgling production was divided by the number of days available for breeding due to survival differences (*r*_s_ = 0.33, p<0.05). The increase in fledgling production was equally due to a higher rate of brood production (i.e. broods produced which resulted in hatchlings) and a larger number of fledglings produced per brood because these components of fledgling production correlated equally with bill colour (rate of brood production: *r*_s_ = 0.295, p = 0.09; fledglings per brood: *r*_s_ = 0.290, p = 0.10). Within males no significant relationships were detected between bill redness and the measures of reproductive success we tested above in females (figure 2 left panel; range *r*_s_ = −0.25 | −0.14, p>0.23). The likelihood of ending up in a pair after introduction was higher for females that exhibited redder bills (Χ^2^(1) = 5.44, p = 0.02, N = 22), but we did not detect such a relationship in males (Χ^2^(1) = 0.96, p = 0.33, N = 25) and within pairs male and female bill hue did not correlate (*r* = 0.08, N = 12, p = 0.81).

## Discussion

Male and female zebra finches with redder bills showed increased survival, in particular among birds with bills that were less red than average (figure 1). In other bird species male sexual ornaments have also been linked to survival, reviewed in [39]. However, for carotenoid dependent traits there are relatively few examples [40]–[43] and evidence is particularly sparse in females with only two published studies that we are aware of [18], [42]. Our findings thus substantiate signalling of phenotypic quality by zebra finch bill colouration. This contradicts an earlier report of females with less red bills showing the highest survival rates [18]. In females, but not in males, we detected that the relation between bill colour and survival levelled off at higher redness (figure S1), with no significant relation among females with the reddest bills. Although we do not detect significant negative selection against redder bills in females it may be suggestive of sexually antagonistic selection revealing itself among the reddest females. This would also fit with the observation of Burley & Coopersmith [33] in which male zebra finches were shown to prefer females with intermediate bill hues.

Females with redder bills also produced more fledglings, contrary to earlier findings [18]. This, together with increased survival of redder females suggests positive selection for bill redness in both males and females, instead of sexually antagonistic selection as reported by Price & Burley (1994) [18]. This discrepancy may be due to several reasons. The first reason may be a matter of sample size and follow up. In Price & Burley’s study the sample size for survival was lower (N = 30 males and N = 30 females vs. N = 72 males and N = 68 females in our study) and follow up was shorter (1.7 years vs. 3.1 years). Second, for their experiment Price & Burley selected the least red and reddest individuals from a larger population. When relationships are non-linear, as we demonstrated for the association between female colouration and survival, the findings will be strongly influenced by the criteria used to select different subsets. Third, survival in Price & Burley’s study was measured under *ad libitum* reproduction, which may affect the relationship of bill colouration with survival. We avoided this issue by studying survival in a setting without reproduction, but for comparability with the study of Price & Burley also tested the association between bill colour and survival among the breeding birds. In the batch of females under *ad libitum* reproduction for which we had the longest follow up (N = 22, 15 deaths, survival follow up: 2.8 years) the associations were similar (linear term: −0.40± S.E. 0.15, p = 0.007; quadratic term: 0.05± S.E. 0.038, p = 0.17) to those we report for single-sex housed females in our survival study. Within males we did not detect significant associations of bill hue with survival (N = 25, 11 deaths, survival follow up: 2.8 years, linear term: 0.14± S.E. 0.13, p = 0.30). Interestingly Price & Burley also found no significant association of bill hue and survival within males contrary to females. This suggests that within males the association between bill hue and survival is lost under *ad libitum* reproduction. In continuing our *ad libitum* reproduction experiment we will increase our sample size to test this hypothesis. Fourth, we cannot exclude the possibility that there are population differences (caused by e.g. husbandry, origin of birds, environmental differences) in the relations we studied.

Given that we found no evidence for sexually antagonistic selection for bill colouration we expected assortative mating instead of possible disassortative mating. In accordance with this expectation we found that redder females were more likely to be engaged in pair formation, possibly mediated by male choice, but in our limited sample we do not find evidence for assortative mating. This may be attributed to assortative mating among extra-pair copulations, which we did not establish in this study. We conclude that bill colouration of male and female zebra finches signals phenotypic quality. This suggests that in both males and females the deposition of carotenoids into bill colouration ensures signal honesty.

## Supporting Information

Figure S1 **Predicted hazard from the model including all females (** **table 3** **).** The predicted relationship is plotted for the range of bill hues observed within this specific set of females. Hazard rate sharply drops when bill hue increases, but levels off and tends to increase at the highest bill hues (see main text).
(PDF)

Table S1 **Presented are the medians per batch of the reproduction measures we analysed, along with the non-parametric test for differences between batches.**
(PDF)
