# Supplementary figures and images for: Bill Redness Is Positively Associated with Reproduction and Survival in Male and Female Zebra Finches
Source: PLoS One. 2012 Jul 12;7(7):e40721. doi: 10.1371/journal.pone.0040721 (PMC3395645; doi:10.1371/journal.pone.0040721)

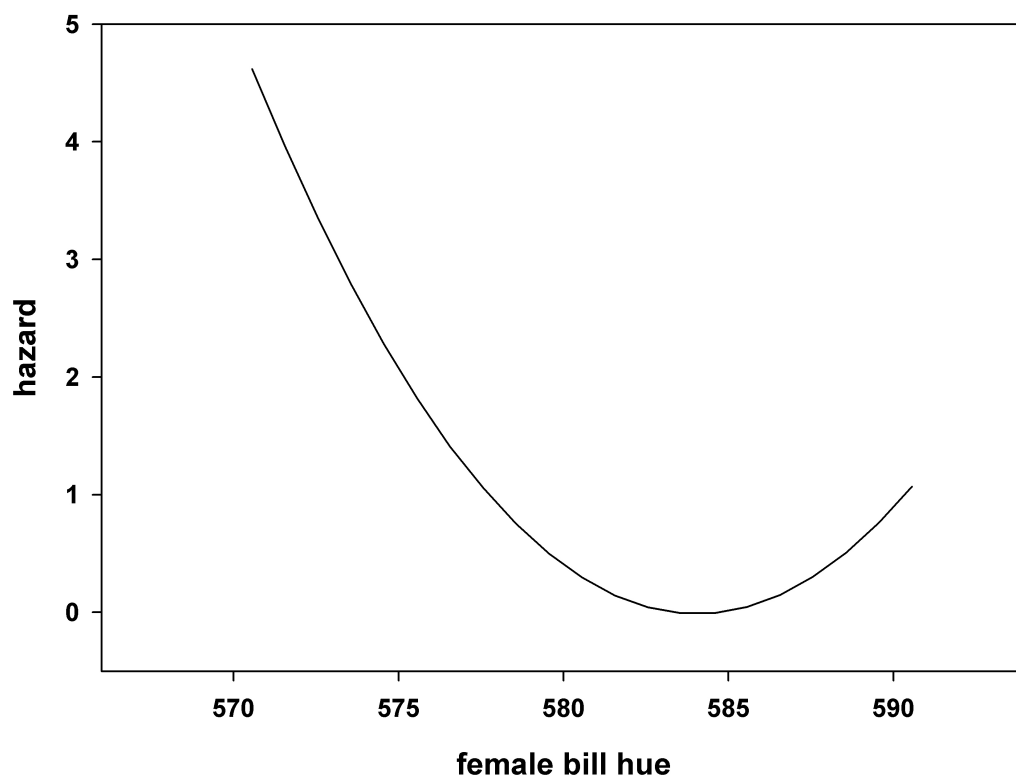

Supplement: Figure S1 — Predicted hazard from the model including all females ( table 3 ). The predicted relationship is plotted for the range of bill hues observed within this specific set of females. Hazard rate sharply drops when bill hue increases, but levels off and tends to increase at the highest bill hues (see main text). (PDF) [file pone.0040721.s001.pdf]
