# Supplementary material for: Bill Redness Is Positively Associated with Reproduction and Survival in Male and Female Zebra Finches
Source: PLoS One. 2012 Jul 12;7(7):e40721. doi: 10.1371/journal.pone.0040721 (PMC3395645; doi:10.1371/journal.pone.0040721)
Supplement: Table S1 — Presented are the medians per batch of the reproduction measures we analysed, along with the non-parametric test for differences between batches. (PDF) [file pone.0040721.s002.pdf]

variable	batch 1	batch 2	Wilcoxon test
**fledging produced in the two seasons of follow up**	4	21	p= 0.0028
**broods (which included hatchlings) produced in the two seasons of follow up**	2	8	p= 0.011
**fledglings per brood**	1.5	3	p= 0.016
